# Effectiveness and safety of a long-acting, once-daily, two-phase release formulation of methylphenidate (Ritalin^®^ LA) in school children under daily practice conditions

**DOI:** 10.1007/s12402-014-0154-x

**Published:** 2014-10-28

**Authors:** Fabian Haertling, Beate Mueller, Oliver Bilke-Hentsch

**Affiliations:** 1 Outpatient Clinic for Child and Adolescent Psychiatry, Frankfurt, Germany; 2 Novartis Pharma GmbH, Nuremberg, Germany; 3 Modellstation SOMOSA, Zum Park 20, 8404 Winterthur, Switzerland

**Keywords:** Methylphenidate, QoL, Treatment practice, Observational study

## Abstract

Long-acting (LA) preparations of methylphenidate allow for once-daily dosing; however, pharmacokinetics may vary and depend on food intake. The objective was to evaluate effectiveness of a two-phase release formulation (Ritalin^®^ LA) under daily practice conditions. This was a prospective, multicenter, observational study in Germany. Eligibility and dosing were determined by the physician based on the drug label. Outcomes included changes over 3 months of treatment in assessments of effect duration, clinical global impression (CGI), and quality of life (ILK). In 101 sites, 262 patients (197 boys, 63 girls, and two unknown) with a mean age of 10.9 years were enrolled; 50 were treated for the first time; 212 switched medication to Ritalin^®^ LA. After 3 months, CGI improved in 59.4 % of patients, and well-being overall was rated as good by 61.0 % of parents and 63.7 % of children. Based on parents’ assessment, the proportion of children suffering from strong disease burden decreased from 40.7 to 15.1 %. In 123 insufficient responders to previous ADHD medications, benefit from Ritalin^®^ LA was above average and effect duration was significantly prolonged as compared to pretreatment. Overall, 28 patients (10.7 %) had treatment-related adverse events with one case being serious; 23 patients (8.8 %) discontinued therapy, 7 (2.7 %) due to poor treatment response; and 212 patients (81 %) continued treatment beyond the study. In line with clinical trial data, Ritalin^®^ LA provides significant benefit also under routine practice conditions.

## Introduction

Attention-deficit hyperactivity disorder (ADHD) is among the most common psychiatric disorders in children and adolescents with an estimated prevalence of 3–5 % (Dopheide and Pliszka 2009; Greenhill 1998; NIH 1998; Scahill and Schwab-Stone 2000). This neurobiological disorder is characterized by significant impairment of concentration and attention, associated with a lack of impulsivity control and increased motor activity (AWMF 2007). Without treatment, these symptoms may persist in adulthood and adversely affect socioemotional and intellectual development (Buitelaar and Medori 2010). Treatment in children is usually multimodal comprising psychotherapy together with occupational therapy, educational training including parents, and eventually drug therapy (Brown et al. 2005; Dopheide and Pliszka 2009; Grosse and Skrodzki 2007; Reddy 2013).

With more than 6,000 patients treated in over 200 clinical trials, methylphenidate (MPH) is one of the best studied drugs in children and adolescents (Vitiello 2001). Based on evidence of its efficacy from several clinical trials and meta-analyses (Faraone et al. 2004; Group 1999; Kutcher et al. 2004; Schachter et al. 2001), MPH has become a mainstay in ADHD therapy and the first-line drug recommendation (AWMF 2007; Grosse and Skrodzki 2007; Reddy 2013). Conventionally, MPH treatment is initiated with immediate release formulations to titrate the dose (Kimko et al. 1999; Kutcher et al. 2004), usually given two to three times daily, depending on the individual effect duration and the severity of symptoms. After the appropriate dose is determined, patients are often switched to a long-acting (LA) formulation taken at home to avoid drug intake in school or in the presence of peers ensuring compliance and steadiness of effects (Grosse and Skrodzki 2007). For these reasons, more recent practice guidelines recommend the use of modified-release formulations even from the outset (National Collaborating Centre for Mental Health 2009). Several modified-release formulations with varying pharmacokinetic (PK) profiles are available, none of which is preferentially recommended by treatment guidelines. Advice given early on to choose the preparation and then to titrate the dose according to individual requirements (AWMF 2007; Banaschewski et al. 2008a, 2008b; Grosse and Skrodzki 2007) is still valid (Coghill et al. 2013).

Due to the Spheroidal Oral Drug Absorption System (SODAS) technology, Ritalin^®^ LA releases 50 % of the drug immediately and 50 % after 4 h. This ensures effect duration of 8–9 h with one single dose, regardless of food intake (Markowitz et al. 2003). Independence from food intake might be important, as children with ADHD have different dietary patterns (Dura Trave et al. 2014), and only about half of them were reported to have breakfast on a regular basis (Oehler 2007). The efficacy and safety of this modified-release formulation has been shown in placebo-controlled (Biederman et al. 2003) and active-controlled clinical trials (Lopez et al. 2003; Schulz et al. 2010; Silva et al. 2005). However, despite high internal validity, clinical trials are known to be prone to selection bias and to not accurately reflect real-life conditions, particularly with regard to compliance, which may considerably improve with easier dosing regimens. Effectiveness in real life can only be investigated by observational studies. Therefore, the present study investigated the effectiveness of Ritalin^®^ LA in an average pediatric ADHD population in Germany over 3 months. The aim was to assess clinical benefits and the effective period/duration of action of treatment with Ritalin^®^ LA.

## Methods

### Study design

This was a multicenter, 12-week, observational study. For each patient, physicians were requested to collect data immediately prior to and about 1 month (optional) and 3 months after treatment initiation. Upon ethics committee approval, the study was conducted from December 2008 to August 2010 in compliance with the Declaration of Helsinki and all applicable legal requirements in Germany. Parents were informed about the study and had to consent to participation of their child and to the analysis of its pseudo-anonymized data. The study was registered on the Web site of the German Association of Researching Pharmaceutical Companies (Verband forschender Arzneimittelhersteller, VfA: http://www.vfa.de/de/arzneimittel-forschung/datenbanken-zu-arzneimitteln/nisdb/nis-details/_484).

### Patients and treatment

As this was an observational study, there were no protocol restrictions regarding eligibility of patients and their treatment. Both were to be decided on by the attending physician based on the drug label. This specifies (1) the drug to be indicated for the treatment of ADHD with hyperactive-impulsive or inattentive symptoms persistent for at least 6 months and having caused clinically significant impairment in at least two settings and (2) the dosage to be adjusted according to the individual needs and responses of patients. It recommends to start with 20 mg once daily and to adjust in weekly 10 mg increments to a maximum of 60 mg/day taken once in the morning. For patients switching from another MPH formulation, it is recommended to maintain the former total daily dose.

### Outcome measures

Effectiveness was evaluated after 3 months of treatment based on: (a) clinical global impression severity (CGI-s) of patients as rated overall and regarding four aspects (attention, impulse control, hyperactivity, and learning problems) on a 4-point scale (inconspicuous, mildly/moderately/severely abnormal) by the attending physician (Dopfner et al. 2006). As this was an observational study in over 100 centers, which rendered dedicated investigator trainings unfeasible, changes in CGI were not rated separately by the attending physician as suggested by Dopfner and colleagues, but calculated as differences of CGI-s scores; (b) patients’ quality of life (QoL) and disease burden based on nine questions of the German inventory for the assessment of QoL in children (ILK). These referred to (1) coping with school demands, (2) relationship to family members, (3) other children, (4) ease with self-employment, (5), physical health, (6) general mood, (7) overall well-being, (8) perceived disease, and (9) therapy burden and were to be evaluated on a 5-point scale (very good, good, intermediate, rather poor, and very poor) by parents and children (Mattejat and Remschmidt 2006).

Further outcome measures were the onset and the end of any subjectively perceived effect as recorded by parents. In addition, parents were asked at each visit whether breakfast of their children over the preceding month had (in their eyes) always been adequate, and whether quantity and quality had varied.

Drug tolerability and safety were evaluated based on adverse event (AE) monitoring at each scheduled visit and treatment practice and compliance based on recordings of dose, dose adjustments, concomitant medication, and premature discontinuation of the study medication.

### Data management and analyses

All data were collected on paper Case Report Forms and managed as well as analyzed by a Contract Research Organisation (SIMW, Wegberg, Germany). Diseases were classified according to the International Classification of Diseases (ICD-10); medications according to the WHO Drug Dictionary as of March 1, 2007; and AEs according to the Medical Dictionary for Regulatory Activities (MedDRA), version 13. Data analysis was mostly descriptive using summary statistics for categorical and quantitative data. Safety analyses included tabulation of type and frequency of AEs, on a patient and event basis. Inferential statistics were only applied for subgroup analyses evaluating outcomes in patients having ceased previous treatment due to poor response (“insufficient responders”) to examine potential benefits added by Ritalin^®^ LA. For this, Fisher’s exact test, Wilcoxon matched pair test, and Mann–Whitney test were used at a significance level of 0.05. All statistical analyses were performed using the software STATISTICA, version 8.0 (StatSoft Inc., Tulsa, USA).

## Results

### Patient population

In total, 262 patients were enrolled in 101 sites across Germany, 197 boys (75.2 %) and 63 girls (24.0 %); for two children, sex had not been documented (0.8 %). Mean age and weight were 10.9 ± 2.5 (SD) years and 41 ± 20 kg, respectively. Disturbance of activity and attention (F90.0) was most commonly diagnosed (58 %), followed by hyperkinetic conduct disorder (F90.1: 34 %) and attention-deficit disorder without hyperactivity (F98.8: 8 %). Fifty patients (19.1 %) had new-onset ADHD and received drug treatment for the first time. The remaining 212 patients (80.9 %) switched to Ritalin^®^ LA, most frequently from Medikinet retard^®^ (*n* = 64); less common premedications were Concerta^®^ (35), Ritalin^®^ (33), Strattera^®^ (19), Equasym^®^ (17), or Equasym^®^ retard (16). The most common reason for changing drug treatment was poor response in 123 patients (58.0 %), followed by a preference for the retarded formulation in 64 patients (30.2 %), and tolerability problems in 37 (17.4 %). At baseline, the subgroup of patients switching to Ritalin^®^ LA for poor effectiveness (“insufficient responders”) was comparable to the remaining population in demographic and disease characteristics, with the exception of a higher percentage of male patients (Table 1).

**Table 1:** Demographics, disease characteristics, and breakfast habits in subgroups of patients with insufficient response to previous treatment and the remaining population

N	Insufficient responders	Remaining population	Total population
	123	139	262
Age [years]	10.9 ± 2.4	10.8 ± 2.6	10.9 ± 2.5
Weight [kg]	40.4 ± 14.7	42.3 ± 23.1	41.4 ± 19.7
Sex			
Male (%)	82.9	68.3	75.2
Female (%)	17.1	30.2	24.0
ICD-10 Diagnosis			
F 90.0 (%)	58.5	57.5	58.0
F 90.1 (%)	36.6	31.7	34.0
F 98.8 (%)	4.9	10.1	7.6
Breakfast adequacy*			
Always (%)	45.5	46.8	46.2
Not always (%)	52.9	51.8	52.3
Breakfast quantity*			
Constant (%)	43.9	32.4	37.8
Varying (%)	51.2	63.3	57.6
Breakfast quality*			
Constant (%)	30.9	28.8	29.8
Varying (%)	56.9	64.0	60.7

* As judged by parents

In comparison with the overall population, the subgroup of patients newly treated with Ritalin^®^ LA (*n* = 50) had a higher percentage of female patients (34 %), were more often diagnosed with an attention-deficit disorder without hyperactivity (16 %), and had less regularly adequate breakfast (62 vs 46.2 % in the total population).

### Effectiveness

#### Assessment of CGI-s by the physician

**Fig. 1:**
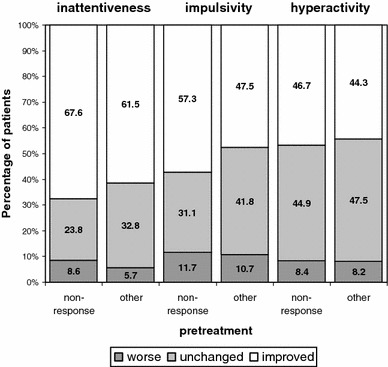
Change in the three aspects of the CGI-s (inattentiveness, impulsivity, and hyperactivity) after 3 months of treatment with Ritalin^®^ LA, as assessed by the investigator, in groups of patients who switched treatment either for “non-response” to previous treatment (*n* = 123) or for “other” reasons (*n* = 139)

At the end of the study, the CGI-s in the total population had improved in 137 (59.4 %) patients (Fig. 1). More patients showed improvement in the area of inattentiveness as compared with impulsivity control and hyperactivity (64.4 vs 51.3 % and 44.8 % of patients, respectively). Learning problems were reduced in 50.4 % of patients and problems due to ADHD in general in 61.1 % of patients.

#### Assessment of QoL and disease burden by parents and children

**Fig. 2:**
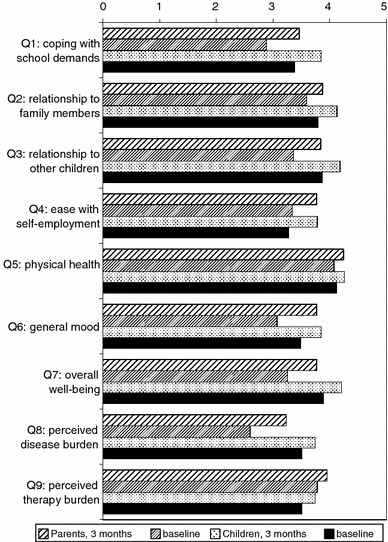
Mean scores (0: very poor to 5: very good) of parents and children on the nine questions of the ILK at baseline and after 3 months of treatment

Mean scores on all nine items of the ILK had slightly improved after 3 months of treatment (Fig. 2). At baseline, parents rated the overall well-being in 101 (38.7 %) and the mental condition in 74 (28.3 %) of the 262 children as good. After 3 months of treatment, both rates increased to 61.0 %. Regarding the good overall well-being, the self-assessment of children matched well (63.7 %), although 54.4 % already felt well at baseline; 39.0 % also rated their mental condition at baseline to be good while in comparison with parents less children did so after treatment (47.7 %). By contrast, the rate of good physical health hardly changed through treatment, neither for parents (77.7 vs 76.3 % at baseline) nor for children (64.1 vs 61.5 % at baseline). Parents rated 40.7 % of children to cope well with the demands at school as compared to 23.5 % at baseline. Corresponding self-assessment of children was slightly higher after treatment (47.0 %) and considerably higher at baseline (36.3 %). According to parents, the percentage of children with a good relationship to family members and to fellows increased from 51.4 to 59.7 % and from 45.1 to 57.7 %, respectively. Corresponding self-assessments of children after 3 months matched well (56.9 and 54.9 % vs 52.7 % at baseline), although changes were smaller. Good ability for self-entertainment had developed in 54.6 % as compared to 44.6 % at baseline, as assessed by parents. Self-assessments of children showed similar changes at a lower level (46.9 vs 36.2 % at baseline).

Parents’ assessment of the disease burden to be strong decreased from 40.7 to 15.1 % of their children and from 44.3 to 19.9 % for themselves. By contrast, only one-third of children perceived the disease burden as strong without any impact of treatment. The burden associated with treatment was generally perceived low already at baseline and hardly changed along with Ritalin^®^ LA, neither for parents nor for children.

Therapy was continued beyond the end of the study in 212 patients, whereas 23 patients (8.8 %) discontinued treatment prematurely, 7 (2.7 %) because of poor treatment response.

#### Effectiveness in subgroups of patients switching from another ADHD medication to Ritalin^®^ LA

Among the patients switching ADHD medication, 59.3 % of the 123 “insufficient responders” perceived a distinct improvement with Ritalin^®^ LA as compared to 48.5 % of 89 patients switching for other reasons. The percentage of patients with any improvement was about the same in both groups (74.4 vs 74.6 %, respectively). The proportions of insufficient responders showing improvements in inattentiveness, impulsivity, and hyperactivity were comparable to the remaining population (Fig. 1), and perceived improvements regarding QoL and disease burden were also about the same (Table 2).

**Table 2:** Change in QoL (Mattejat and Remschmidt 2006) for the subgroups of insufficient responders to previous treatment and the remaining population over 3 months of treatment with Ritalin^®^ LA

	A: insufficient responders	B: remaining population	P*
Parents	−5.0 ± 5.6 (69)	−4.2 ± 6.1 (80)	0.1875
Children	−4.1 ± 5.3 (36)	−3.4 ± 6.5 (51)	0.6040

Values are mean ± standard deviation (number of patients with data)

* Mann–Whitney test

The proportion of “insufficient responders” was particularly high (73.4 %) in the largest subgroup of patients pretreated with Medikinet retard^®^. Under Ritalin^®^ LA, the CGI-s of these patients improved more often than average (64.3 vs 59.4 % in the total patient population; inattentiveness: 67.3 vs 64.4 %; impulsivity: 60.7 vs 51.3 %; and hyperactivity 44.6 vs 44.8 %, respectively). A similar trend was observed with QoL scores of parents and children. Ratings from parents showed an improvement after 12 weeks on all questions, most notably global impression and psychological state/mood (47 and 48 % with improvement, respectively). Self-assessment of children also revealed an improvement after 12 weeks on all questions, including the global impression (43 %) and psychological state/mood (60 %).

Ritalin^®^ LA showed a significantly prolonged duration of effect perceived by parents as compared to the preceding MPH medications (Table 3). This prolongation was more pronounced in insufficient responders as compared to those changing for other reasons (on average, 1.4 versus 0.5 h, Table 3). There was also less variation in effect duration with Ritalin^®^ LA.

**Table 3:** Effect duration [h] of the preceding MPH medication and Ritalin^®^ LA, as assessed by parents at the baseline and the final visit, respectively

	Preceding MPH	Ritalin^®^ LA	P*
All changing patients	6.2 ± 2.0 (147)	7.3 ± 1.4 (224)	<0.001
Subgroups changing for			
Poor effectiveness	6.1 ± 2.0 (87)	7.5 ± 1.4 (103)	<0.001
Other reasons	6.5 ± 2.0 (60)	7.0 ± 1.4 (119)	0.037
P**	0.139	0.023	

Values are mean ± standard deviation (number of patients with data)

* Wilcoxon; ** Mann–Whitney test

### Safety

A total of 63 AEs were reported in 36 (13.7 %) patients. In three patients, AEs were considered serious due to hospitalization, one because of sleepiness, decreased appetite, and activity, one for insufficient treatment response, and one for unknown reasons. The latter two were thought to be unrelated to treatment. Physicians deemed 46 of the non-serious AEs at least possibly related to MPH. These potentially related AEs occurred in 28 patients (10.7 %) of whom 12 patients had one, 10 patients two, five patients three, and one patient four such AEs. Overall, incidence rates of potentially MPH-related and serious adverse events were 10.3 and 0.4 % of patients, respectively. The most frequent AEs were loss of appetite, abdominal pain, and nausea, the most frequently affected System Organ Classes (SOCs) were metabolism and nutritional as well as psychiatric and nervous system disorders (Table 4). Severity was mild in 30.2 %, moderate in 39.7 %, and severe in 22.2 % of AEs; 7.9 % of data were missing. By the end of the study, more than half of the AEs had resolved completely.

**Table 4:** Incidence of adverse events (AEs) and serious adverse events (SAEs) in 28 patients that physicians deemed at least possibly related to MPH (adverse drug reactions, ADR), by System Organ Class and Preferred Term

MedDRA system organ class	AE		SAE		Total	
Preferred term	*N*	%	*N*	%	*N*	%
Total of patients	262	100	262	100	262	100
Patients with ADR	27	10.3	1	0.4	28	10.7
Metabolism and nutritional disorders	11	4.2	1	0.4	12	4.6
Anorexia	11	4.2	1	0.4	12	4.6
Nervous system disorders	10	3.8	0	0.0	10	3.8
Dizziness	1	0.4	0	0.0	1	0.4
Agitation	2	0.8	0	0.0	2	0.8
Confusional state	1	0.4	0	0.0	1	0.4
Headache	2	0.8	0	0.0	2	0.8
Insomnia	3	1.1	0	0.0	3	1.1
Paralysis	1	0.4	0	0.0	1	0.4
Psychiatric disorders	9	3.4	1	0.4	10	3.8
Aggression	3	1.1	0	0.0	3	1.1
Depression	1	0.4	0	0.0	1	0.4
Affective disorder	1	0.4	0	0.0	1	0.4
Dysphemia	1	0.4	0	0.0	1	0.4
Mood swings	1	0.4	0	0.0	1	0.4
Emotional disorders of childhood	1	0.4	0	0.0	1	0.4
Sleep disorder	1	0.4	1	0.4	1	0.4
Gastrointestinal disorders	8	3.1	0	0.0	8	3.1
Nausea	4	1.5	0	0.0	4	1.5
Abdominal pain	4	1.5	0	0.0	4	1.5
General disorders & administration site conditions	2	0.8	1	0.4	3	1.1
Decreased activity	0	0.0	1	0.4	1	0.4
Rebound effect	1	0.4	0	0.0	1	0.4
Asthenia	1	0.4	0	0.0	1	0.4
Investigations	3	1.1	0	0.0	3	1.1
Weight decreased	3	1.1	0	0.0	3	1.1
Skin and subcutaneous tissue disorders	1	0.4	0	0.0	1	0.4
Rash	1	0.4	0	0.0	1	0.4
Respiratory, thoracic and mediastinal disorders	1	0.4	0	0.0	1	0.4
Chest discomfort	1	0.4	0	0.0	1	0.4
Social circumstances	1	0.4	0	0.0	1	0.4
Educational problem	1	0.4	0	0.0	1	0.4

## Discussion

This observational study aimed at evaluating benefits offered by Ritalin^®^ LA to children with ADHD under routine practice conditions in Germany. It demonstrated improvements in ADHD symptoms and QoL, confirmed overall good tolerability and safety, and revealed a longer perceived effect duration versus previous treatments. Comparable results regarding safety and effectiveness of other sustained release MPH formulations in daily practice have already been reported, particularly after switching from immediate release formulations (Dopfner et al. 2011a, 2011b; Rothenberger et al. 2011). However, to our knowledge, a significant prolongation of the perceived effect under such conditions is described here for the first time, although recently a change in the daily course of ADHD symptoms after switching to another LA formulation has been reported under routine practice conditions too (Froelich et al. 2014). Moreover, the study confirmed preliminary data indicating children with ADHD to often have irregular breakfast (Dura Trave et al. 2014; Oehler 2007) and overall the assessments of children and parents regarding the impact of changes in ADHD symptoms on QoL to match well.

Differences in effectiveness and in particular of effect durations may be linked to different PK profiles exhibited also by sustained release formulations (Auiler et al. 2002; Banaschewski et al. 2008a; Coghill et al. 2013; Gonzalez et al. 2002; Haessler et al. 2008; Liu et al. 2005; Lopez et al. 2003; Markowitz et al. 2003). Medikinet^®^ retard and Ritalin^®^ LA were shown not to be bioequivalent in fed state, and this difference in PK profiles was assumed to be of clinical relevance (Fleischhaker et al. 2010; Haessler et al. 2008). PKs and effect duration of some sustained release formulations including Medikinet retard^®^ are known to vary with food intake (Midha et al. 2001), whereas release patterns of others including Ritalin^®^ LA are unaffected (Lee et al. 2003). In fact, the current study confirmed children with ADHD to have irregular breakfast habits (Oehler 2007). However, due to limitations mostly inherent to the observational design, it can not conclusively be answered whether the observed longer perceived effect duration of Ritalin^®^ LA was actually resulting from its specific PK characteristics. No PK measurements could be performed, and subgroups were too small and heterogenous for a comparison with each individual LA premedication. Short-acting MPH formulations may have contributed to the shorter effect duration of premedication in the whole “insufficient responder” group. Finally, results on effect duration and on breakfast adequacy were based on subjective assessments only which are prone to recall bias. However, both subjective assessments as well as recall bias should have affected the current and the preceding medication in the same way and may thus explain the spread of results rather than the difference between treatments. In terms of safety, AEs are generally known to be underreported in observational studies. However, despite those limitations, overall, our data are still supporting effectiveness and tolerability of Ritalin^®^ LA in daily practice. Controlled clinical trials are warranted to identify subgroups of patients who might benefit most.

## Conclusion

Ritalin^®^ LA improved CGI and QoL in children with ADHD under routine practice conditions. Results were in line with those from controlled clinical trials although further studies will be needed to investigate differences in perceived effect durations.
